# Health Problems in Individuals With PWS Are Associated With Lower Quality of Life for Their Parents: A Snapshot in the Brazilian Population

**DOI:** 10.3389/fped.2022.746311

**Published:** 2022-02-15

**Authors:** Alexandre Slowetzky Amaro, Daniela Andrea Rubin, Maria Cristina Triguero Veloz Teixeira, Arcenio José Ferreira, Graciele Massoli Rodrigues, Luiz Renato Rodrigues Carreiro

**Affiliations:** ^1^Development Disorders Graduate Program, Center for Health and Biological Science, Universidade Presbiteriana Mackenzie, São Paulo, Brazil; ^2^Department of Kinesiology, California State University, Fullerton, CA, United States; ^3^Physical Education Program, Universidade São Judas Tadeu, São Paulo, Brazil; ^4^Physical Education Program, Escola Superior de Educação Física de Jundiaí, Jundiaí, Brazil

**Keywords:** Prader-Willi syndrome, obesity, treatment, quality of life, behavior problems

## Abstract

Prader-Willi syndrome (PWS) is a complex genetic disorder requiring interdisciplinary team monitoring and intensive care by parents. So far there is little information on people with PWS in Brazil. Our aim was to describe health problems and treatments used by people with PWS in Brazil and their relationship to their parents' quality of life. Parents answered questionnaires about their child's medical and exercise history, behavior problems, sociodemographic characteristics, and their own quality of life. Results: The responses of the participants showed similar health problems as in other countries. Anxiety and tantrums were the behavioral problems most commonly cited by parents. Parents of people with PWS had lower scores in respect of quality of life than the Brazilian population. Behavioral problems in individuals with PWS were negatively associated with their parents' quality of life. Behavioral and medical conditions in the children were associated with reduced quality of life in the parents. We conclude that heath care should not only be directed toward those with PWS, but also their parents.

## Introduction

First described by Prader, Labhart, and Willi in 1956, Prader-Willi syndrome (PWS) is the result of the absence of gene expression of the q11.2-q13 region from chromosome 15 of paternal origin. PWS affects 1:20,000 births and has a prevalence of 1:54,000 (1), and is usually caused by one of three genetic mechanisms: deletion of the paternally inherited 15q11.2-q13 region (DEL), maternal uniparental disomy (UPD), or an imprinting defect (ID) (2, 3). PWS presents a complex clinical and psychiatric phenotype with variations between its genetic subtypes that produce a range of symptoms that include severe neonatal hypotonia, growth hormone deficiency (4), poor motor skills (5), and mild to moderate cognitive deficits (6). Hyperphagia and severe obesity are the main clinical features of the syndrome (7). Hyperphagia begins at about 8 years old and reaches its apex in late adolescence and remains stable throughout life (8). Hyperphagia leads the person with PWS to seek food constantly because the individuals rarely feel full (2, 9).

The most common non-pharmacological strategies used to maintain a healthy weight in PWS include the strict control of caloric intake and the access to food, and exercise (10). However, if these strategies are implemented without adequate medical and nutritional monitoring and parental support, there can be a significant increase in maladaptive behaviors that can become a major source of family stress (11, 12). The most common of these maladaptive behaviors are food-seeking behaviors, severe emotional and behavioral outbursts, hyperphagia, tantrums, stubbornness, manipulative behavior, obsessive-compulsive behaviors, heightened anxiety, sleep disorders, self-injury behaviors, cognitive rigidity, and psychotic symptoms in adulthood (13–15).

Pharmacological treatments are commonly employed to manage the complex clinical conditions of PWS. Psychotropics are prescribed for behavior problems and neuropsychiatric disorders; laxatives for constipation problems; dermatological formulas for the treatment of the ulcerations due to constant skin picking; and antidiabetic and hypotensive drugs for the comorbidities related to obesity (16). However, for the management of hyperphagia several drugs are under study at different phases of clinical trials (10). Hormonal treatment is also extensively employed in PWS, especially growth hormone replacement therapy (GHRT) which is part of the standard of care in addition to nutrition and exercise (10). Studies have shown the benefits of GHRT in respect of psychomotor development (17), cognitive and language function (18, 19), growth in children and adolescents (20), and improved body composition (21, 22). Physical activity and exercise are highly recommended in PWS (23). Increased levels of physical activity have been associated with improved weight and body composition, muscular strength (24, 25), motor skills (25, 26), and metabolic markers related to health (26).

It is a consensus among PWS experts that early intervention and an interdisciplinary approach are the best practices to maintain the health of people with PWS (2, 27). However, the routine of visits to different specialists, drug treatments, and the management of hyperphagia and behavioral problems can overwhelm parents with a high level of physical and mental stress, leading them to a severe decline in their health and quality of life (3, 28–31).

The relationship between physical and mental health problems in children with PWS, treatments and behavior problems, and the quality of life of the parents of individuals with PWS is not well-established. A better understanding of the associations between these domains could contribute to improved intervention programs for patients with PWS and their parents. This study aimed to: (1) describe health conditions and behavioral problems in Brazilian children, adolescents and adults with PWS and (2) to examine the possible associations between health conditions and behavioral problems in individuals with PWS and their parents' quality of life.

## Materials and Methods

### Participants

The sample included 41 individuals (children, adolescents, and adults) aged from 1 month to 38 years with genetically confirmed PWS. The patients were recruited mainly from the database of the Graduate Program in Developmental Disorders of Mackenzie Presbyterian University, medical genetics centers of other universities, and from Brazilian PWS social networks. The primary caregiver (parent or guardian) had to be aged over 18 years and had to provide genetic diagnosis for their child or the person with PWS they cared for.

### Materials and Measures

In order to collect the data of this study, we used:

#### Sociodemographic Questionnaire

For characterization of the sociodemographic profile of the sample comprising items that included sex, civil status, level of education, and type of health insurance.

#### Medical History and Exercise Questionnaire

A questionnaire was completed that included a medical and exercise history developed by Rubin et al. (32) and translated into Brazilian Portuguese and adapted for this study by the authors (unpublished data). The questionnaire comprises dichotomous questions for the presence or absence of diseases, causes of hospitalization, causes of surgeries, treatments or therapies used, types of physical activity practiced, and total weekly physical activity in number of hours/week. Parents or caregivers also reported the body mass (in kg) and height (in m) of their child measured in the last month. The reported body mass and stature were used to calculate body mass index [BMI; body mass (kg)/height squared (m^2^)]. BMI was then converted to BMI percentile, according to the World Health Organization guidelines (33). For those participants who were over 19 years old, the reference values for the Brazilian population were used to calculate the BMI percentile (34). All items of this questionnaire were based on parental answers.

#### Brief Problem Monitor for Parents (BPM-P 6/18)

This is a short version of the Child Behavior Checklist (CBCL 6/18) (35) that evaluates emotional and behavioral problems of children and adolescents between 6 and 18 years old in three scales: internalizing behavioral problems, externalizing behavioral problems, and inattention problems. Externalizing problems are behaviors such as impulsivity, nervousness, aggressiveness, impatience/restlessness, destructiveness, disobedience, teasing behavior, and fights. Internalizing problems include withdrawal, depression, anxiety, irritability, sadness, somatic complaints related to emotional functioning, shyness, excessive preoccupation, insecurity and fears (35). The BPM-P contains 19 self-reported questions scored on a 3-point Likert scale (0–2 scale), being: 0 = “Not true”; 1 = “Somewhat true” and; 2 = “Very true.” The Achenbach System of Empirically Based Assessment (ASEBA) software generates a *T* score for each variable, indicating the degree of deviance from normality, categorizing the participant as normal or elevated. In this study, parents of children under 6 years old and adults over 18 years old did not answer the BPM-P, since the version used only applies to 6–18 years old. This scale was used to obtain information from the perspective of the parents of the individual with PWS.

#### World Health Organization Quality of Life Instrument Version Brief (WHOQOL-Bref)

This is a comprehensive tool used to measure the self-perception of quality of life. The WHOQOL-bref is composed of 24 questions on a Likert scale, providing the perception of the quality of life in four domains: physical, psychological, social relations, and environment. Additionally, two questions about overall and general health are included (36). We used the Microsoft Excel worksheet developed by Pedroso (37) to calculate the output of each of the domains. This questionnaire was used to evaluate the quality of life of the parents of individuals with PWS.

### Procedures

This study was first approved by the Ethics Committee of Mackenzie Presbyterian University under Code: CEP/UPM n° 784.029/08/2014 and CAEE: 34649314.2.0000.0084). The data collection occurred in three ways: (1) Parents were invited to visit the Developmental Disorders Department of Mackenzie Presbyterian University; (2) The research team visited parents in their homes; (3) The research team conducted the visit with parents remotely using telephone calls and/or video conferences. If the visit took place by phone or by video conference, the consent form was sent by e-mail and parents returned it *via* mail together with a copy of the genetic test confirming the diagnosis.

### Statistical Analysis

This was a descriptive, exploratory, and transversal study. Data are presented as raw and percentage values. Normal distribution for continuous variables was tested using skewness and kurtosis, and comparison tests between sex and genetic subtypes were done using *t*-student and Mann–Whitney tests. Behavior characterization was analyzed in individuals ages 6–18 years and further evaluated with regards to the use of GHRT. We codified the classification of quality of life of parents based on Cruz et al. (38) and used the paired Student's *t*-test to compare the quality of life values with normal reference values according Cruz et al. (38). Spearman correlation (*r*_*s*_) analyses were calculated to assess the associations between the emotional and behavioral problems, age and physical and mental indicators of the individuals with PWS and the quality of life of their parents. Other associations evaluated included those between family monetary income and child's behavior and parents' quality of life. The correlation coefficient were interpreted as: 0.1–0.3, weak; 0.4–0.6, moderate; 0.7–09, strong. The significance level adopted was *p* ≤ 0.05. Therefore, all intervals in the present study were constructed with 95% statistical confidence. However, the *p* > 0.05 and *p* <0.1 were also analyzed because they are close to the acceptance limit (up to 5 percentage points above the alpha value adopted).

## Results

### Sample Description

Table 1 shows the characterization of the parents of individuals with PWS by sexes. Most parents who participated were female, married, with an average income of R$ 7.488,06 (~US$ 2,300.00) (*n* = 31, 25th = 2,000.00, 50th = 4,000.00, 75th = 12,000.00). The age of parents did not differ between sex [*t*_(39)_ = 1.76, *p* = 0.87, *IC* (95%) = −0.92 to 13.32].

**Table 1:** Characterization of parents' profile.

				**Age**	
		***N* = 41**	**(%)**	**Mean**	**SD**
Sex	Male	6	(14.6)	47.17	11.23
	Female	35	(85.4)	40.97	7.37
		***N*** **=** **41**	**(%)**		
Civil status	Married	35	(85.4)		
	Divorced	4	(9.8)		
	Single	2	(4.9)		
Level of education	Below college	18	45.1		
	Above college	21	54.9		
		***N*** **=** **38**	**(%)**		
Health insurance	Private	29	(76.3)		
	Public	9	(23.7)		

Table 2 shows the sociodemographic and clinical characterization of the participants with PWS. There were no statistical differences between female and male participants in respect of age (*U* = 150.50, *p* = 0.157). Body mass and height were reported for 35 participants with PWS (85.37%). The analyses of the BMI percentile showed that 57.1% of participants (*n* = 20) were obese, 8.6% (*n* = 3) were overweight, 28.6% (*n* = 10) were of normal weight and 5.7% (*n* = 2) were underweight. There was no difference in the BMI percentile between participants' sex (*U* = 131.00, *p* = 0.515). Fourteen participants were presently or had been on GHRT.

**Table 2:** Sociodemographic and clinic profile of PWS children, adolescents, and adults.

		***N* = 41**	**(%)**					
**Sex**	Male	24	(58.5)					
	Female	17	(41.5)					
**PWS subtypes**	Deletion	24	(58.5)					
	Maternal uniparental disomy	15	(36.6)					
	Micro-deletion or mutation IC	2	(4.9)					
**Age**		**Mean**	**SD**	**Range**		**Percentile**		
				**Lower**	**Upper**	**25th**	**50th**	**75th**
		11.62	7.9	0.1	38	6.00	11.03	16.02
	Male	13.28	9.18	0.1	38			
	Female	9.28	4.97	2.02	21			
**BMI percentile**		**Mean**	**SD**	**Range**		**Percentile**		
*N* = 35				**Lower**	**Upper**	**25th**	**50th**	**75th**
		81.31	27.61	3	99	71	97	99
	Male, *n* = 20	76.55	30.33	3	99			
	Female, *n* = 15	87.67	22.95	15	99			

*N, Number; PWS, Prader-Willi Syndrome; BMI, Body Mass Index; SD, Standard Deviation; IC, Imprinting Center*.

The number of hospitalizations did not differ as a function of PWS subtypes (DEL and UPD) (DEL *M* = 3.54, *SD* = 2.64, UPD *M* = 3.47, *SD* = 3.78, *U* = 0.152, and *p* = 0.411). The main reasons for hospitalization were cryptorchidism, adenoidectomy, tonsillectomy, and inguinal hernia. The percentage of surgery cases related to cryptorchidism and gynecoplasty was corrected by the number of participants and gender (see Table 3).

**Table 3:** Main surgery cases, frequency of behavioral problems, therapies used, physical activities problems, and practices.

**Numbers and types of surgeries**			**Complaints behavior problems**			**Therapies currently engaged by a participant**			**Main problems that limited adherence to physical activity**			**Main motor activity practices**		
	***N* = 41(%)**			***N* = 41(%)**			***N* = 41(%)**			***N* = 32(%)**			***N* = 32(%)**	
Cryptorchidism (M, *n* = 35)	10	24.39	Anxiety	29	70.73	Speech therapy	25	60.98	Coughing	8	25	Walking	11	34.38
Adenoidectomy and tonsillectomy	6	14.63	Tantrums	23	56.1	Dietary management	24	58.54	Irregular breathing	7	21.88	Gym class	7	21.88
Inguinal hernia	3	7.32	Foul language	22	53.66	Psychological therapy	19	46.34	Difficulty breathing	5	15.63	Interactive games (Wii/DDR)	6	18.75
Laparoscopy of the gallbladder	2	4.88	Skin picking	19	46.34	Growth hormone therapy	14	34.15	Irregular heartbeat	3	9.38	Swimming	6	18.75
Arm fracture	2	4.88	Screaming/yelling	16	39.02	Physical therapy	12	29.27	Wheezing	3	9.38	Dancing	5	15.63
Column	2	4.88	Aggressive/violent actions	14	34.15	Structured physical activity	12	29.27	Chest pain	2	6.25	Soccer	4	12.5
Genioplasty (*F, n* = 17)	1	2.44	Cries easily	14	34.15	Aquatic therapy	11	26.83	Inability to keep up with other children	2	6.25	Strengthening	4	12.5
Femur fracture	1	2.44	Compulsive ordering/counting	11	26.83	Occupational therapy	11	26.83	Dizziness	1	3.13	Bicycling	3	9.38
Percutaneous closure of interatrial communication	1	2.44	Threatening to hurt others	6	14.63	Learning disability services	11	26.83				Stretching or yoga	3	9.38
			Destructive behavior	4	9.76	Horse therapy	2	4.88				Running	3	9.38
			Throwing objects	4	9.76							Jump rope	1	3.13
**Prevalence of comorbidities in people with PWS in Brazil**														
**Cardiorespiratory**			**Neurologic**			**Endocrine/Metabolic**			**Musculoskeletal**			**Others**		
	***N*** **=** **41(%)**			***N*** **=** **41(%)**			***N*** **=** **41(%)**			***N*** **=** **41(%)**			***N*** **=** **41(%)**	
Bronchiolitis	11	26.83	Hypersomnolence	9	21.95	Hypercholesterolemia	7	17.07	Scoliosis	15	36.59	Cryptorchidism	10	24.4
Cardiac anomalies	7	17.07	Hyperthermia	6	14.63	Hypertension	6	14.63	Equinovarus foot	5	12.2			
Asthma	7	17.07	Seizure	5	12.2	Hyperthyroidism	6	14.63	Dysplasia	4	9.76			
Obstructive sleep apnea	3	7.32	Hypothermia	4	9.76	Hypertriglyceridemia	4	9.76	Fracture	3	7.32			
			Angry behavior	2	4.88	Hypothyroidism	5	12.2	Strabismus	2	4.88			
						Diabetes 1	3	7.32	Dislocation of the elbow	2	4.88			
						Diabetes 2	2	4.9						

Table 3 summarizes data related to surgeries, behavioral problems, therapies used, physical activity and comorbidities. The mean incidence of emotional and behavioral problems was 3.2 (*SD* = 3.2, lower = 0, upper = 11) and there were no differences among the PWS subtypes [*t*_(37)_ = 0.994, *p* = 0.327] or sex [*t*_(39)_ = −0.872, *p* = 0.389]. Forty-six percent of parents (46.9%, *n* = 15) believed that the emotional and behavioral problems of their children and adolescents could interfere in physical activity program adherence. There were no differences in the number of therapies engaged in among PWS subtypes [*t*_(39)_ = −0.809, *p* = 0.403] or sex [*t*_(37)_ = 0.495, *p* = 0.623]. The main barriers to physical activities reported by parents were coughing and irregular breathing. The mean number of physical activities practiced was 1.78 (*SD* = 1.50, lower = 0, and upper = 7) and the mean time spent in physical activity was 110.78 (*SD* = 121.91, lower = 0, and upper = 450) minutes per week.

We assessed the behavior problems of participants aged between 6 and 18 years old (*n* = 18, *M* = 10.38, and *SD* = 3.13). It was observed that 38.9% (*n* = 7) reached the internalizing clinical level; 66.7% (*n* = 12) reached the attention clinical level, and 77.8% (*n* = 14) reached the externalizing problems clinical level. In respect of the total score, 77.8% (*n* = 14) of all participants reached a clinical level. Participants who had been on GHRT in the past or were presently on GHRT presented lower externalizing scores (*U* = 15, *p* = 0.035) and a tendency to higher parental quality of life in respect of the environment domain [*t*_(38)_ = 1.824, *p* = 0.076] in comparison to participants who had not been on GHRT.

Table 4 shows the parents' quality of life. The results revealed that parents of people with PWS present significantly lower quality of life in the psychology, social relation and environment domains compared to Brazilian reference values.

**Table 4:** Results of parents' quality of life (WHOQOL).

	***N* = 40**	**Sample value**		**Confidence interval**		**Reference value**				**Confidence interval**	
**Domain**		**Mean**	**SD**	**Lower**	**Upper**	**Mean**	**SD**	***t*** ***(df, 39)***	***p***	**Lower**	**Upper**
Physical		60.91	10.64	36.57	77.71	58.9	10.5	1.197	0.238	−1.39	5.42
Psychological		60.33	8.12	45.33	80	65.9	10.8	−4.337	**<0.001**	−8.16	−2.97
Social relation		60.53	11.56	32	80	76.2	18.8	−8.574	**<0.001**	−19.362	−11.970
Environment		56.75	9.64	32	72	59.9	14.9	−2.067	**0.045**	−6.232	−0.068
Self-assessment		58.8	10.95	24	80						
Total		59.29	8.18	37.54	73.85						

*SD, standard deviation; df, Degree of freedom. Bold font indicates statistical significance below 0.05 (p < 0.05)*.

### Relationships Between Characteristics in Individuals With PWS

There were significant positive and moderate correlations between the age of the person with PWS and number of hospitalizations (*r*_*s*_ = 0.466, *p* = 0.002) and number of behavioral problems presented (*r*_*s*_ = 0.399, *p* = 0.01). There was also a negative correlation between BMI percentile and the number of behavior problems presented by people with PWS (*r*_*s*_ = 0.510, *p* = 0.002).

The age of the individuals with PWS also showed positive and moderate correlations with total number of hospitalization (*r*_*s*_ = 0.466, *p* = 0.002), and total number of behavior problems (*r*_*s*_ = 0.399, *p* = 0.01), externalizing problems (*r*_*s*_ = 0.493, *p* = 0.038), and a marginal correlation with internalizing problems (*r*_*s*_ = 0.467, *p* = 0.051). Additionally, total number of hospitalizations also showed a positive correlation with total score for behavior problems (*r*_*s*_ = 0.479, *p* = 0.044), internalizing problems (*r*_*s*_ = 0.483, *p* = 0.042), and marginal correlations with attention (*r*_*s*_ = 0.418, *p* = 0.084) and externalizing problems (*r*_*s*_ = 0.437, *p* = 0.07).

### Association Between Family Income, Parent's Quality of Life, and Child Profile

There were significant moderate associations between family income and parents' quality of life social domain (*r*_*s*_ = 0.365, *p* = 0.043), environment domain (*r*_*s*_ = 0.469, *p* = 0.008), self-assessment domain (*r*_*s*_ = 0.427, *p* = 0.017), and total (*r*_*s*_ = 0.371, *p* = 0.040). The family income was also associated with the total number of therapies the person with PWS was engaged in (*r*_*s*_ = 0.414, *p* = 0.021). The income also presented a marginal negative and moderate correlation with the number of behavior problems exhibited by people with PWS (*r*_*s*_ = −0.325, *p* = 0.075).

Table 5 shows the associations between parents' quality of life and child demographics, medical history and behavior. The total score for quality of life of parents of persons with PWS was moderately associated with the age of the child (*r*_*s*_ = −0.467), BMI percentile of the child (*r*_*s*_ = −0.353, *p* = 0.037), and total number of hospitalization (*r*_*s*_ = −0.483). Total quality of life was also related to the child's behaviors such as internalizing behavioral problems (*r*_*s*_ = −0.500), externalizing behavioral problems (*r*_*s*_ = −0.557), total behavioral problems (*r*_*s*_ = −0.647).

**Table 5:** Correlation test for parents' quality of life (WHOQOL) and BPM-P indexes and MHEQ survey.

**WHOQOL DOMAIN**		**Participants'** **age**	**Parent's** **age**	**BPM INT**	**BPM ATT**	**BPM EXT**	**BPM total**	**Number of hospitalization**	**Number of behavior problems**	**Number of therapies**	**Level of physical activity**
Physical	*r_*s*_*	−0.039	0.146	−0.364	−0.101	−0.035	−0.054	−0.145	−0.293	0.042	−0.208
	*p*	0.811	0.362	0.137	0.690	0.890	0.832	0.366	0.063	0.792	0.252
Psychological	*r_*s*_*	−0.012	0.061	−0.213	0.022	−0.268	−0.027	−0.184	**−0.355**	−0.067	−0.319
	*p*	0.942	0.703	0.396	0.930	0.281	0.915	0.249	**0.023**	0.676	0.075
Social Relationship	*r_*s*_*	0.041	0.023	−0.459	−0.164	−0.453	−0.308	−0.140	**−0.396**	0.054	0.029
	*p*	0.800	0.884	0.055	0.516	0.059	0.213	0.382	**0.010**	0.738	0.874
Environmental	*r_*s*_*	**−0.314**	−0.025	**−0.510**	−0.097	**−0.575**	−0.342	−0.171	**−0.421**	0.132	−0.046
	*p*	**0.046**	0.877	**0.031**	0.702	**0.013**	0.165	0.285	**0.006**	0.411	0.801
Self-assessment	*r_*s*_*	−0.264	−0.059	−0.253	−0.340	−0.343	−0.254	−0.241	−0.304	0.236	−0.123
	*p*	0.100	0.720	0.311	0.167	0.164	0.309	0.134	0.056	0.143	0.510
Total Score	*r_*s*_*	−0.171	0.021	**−0.500**	−0.025	−0.348	−0.164	−0.204	**−0.440**	0.058	−0.239
	*p*	0.286	0.897	**0.035**	0.922	0.156	0.515	0.201	**0.004**	0.717	0.188

*BPM, Brief Problem Monitor for Parents; INT, Internalizing problems; ATT, Attention problems; EXT, Externalizing problems; r_s_, Spearman correlation coefficient. Bold font indicates statistical significance below 0.05 (p < 0.05)*.

The parents' quality of life in respect of the physical domain was moderately associated with the child's BMI (*r*_*s*_ = −0.332, *p* = 0.051) and the number of the child's behavior problems (*r*_*s*_ = −0.317). The psychological domain was associated with the child's BMI (*r*_*s*_ = −0.377, *p* = 0.026), and number of behavioral problems (*r*_*s*_ = −0.379). The social domain was associated with the child's BMI *r*_*s*_ = −0.355, *p* = 0.036). The quality of life environment domain was associated with the child's age (*r*_*s*_ = −0.402), internalizing (*r*_*s*_ = −0.510), externalizing (*r*_*s*_ = −0.575) problems, and total behavior problems (*r*_*s*_ = −0.455). The self-assessment domain showed a marginal negative correlation with the age of the children (*r*_*s*_ = −0.264) and the number of behavioral problems (*r*_*s*_ = −0.304; Table 5).

## Discussion

We report a descriptive analysis of medical, behavioral and emotional problems in Brazilian individuals with PWS and the associations with their parents' quality of life. People with PWS frequently have restricted access to health services in developing countries such as Brazil (39), often only receiving interventions for managing symptoms without a multidisciplinary approach that includes controlling food access, hormone replacement therapies, special education, and psychological interventions.

The mean BMI percentile was 81.31%, characterizing the sample as obese. This is twice as high as that in a study by Diene et al. (40) of a young French PWS sample, and very similar to results in adults reported by Grugni et al. in an Italian study (41). It is clear that the management of nutrition in people with PWS in Brazil needs to be considered very carefully. Brazilians still eat a large number of high-calorie items low on nutritional value. Additionally, a deficit in therapies and GHRT may help to explain the obesity prevalence in this study, as only 14 participants were or had been on GH. Because of the potential progression of health complications with increased obesity and age, the Brazilian population with PWS may be in a vulnerable position in respect of a premature decline in health.

The health problems reported in this study, which can lead to hospitalization, are also found in other studies of people with PWS (42). In our study, 85.4% of the group had endocrine/metabolic problems, while 68.3% had cardiorespiratory problems, and 63.5% had neurological problems. All of these conditions are a high-risk factor for death in PWS. A study conducted by Tauber et al. (43) analyzed the obituary of 64 people with PWS and found that respiratory diseases caused 61% of deaths. In adults with PWS, respiratory infections and obesity-related comorbidities that affect the cardiorespiratory system increase the mortality rate for people over 30-years of age. Among children with PWS, respiratory infections accompanied by a high fever and a lack of immunological response to medication are the major mortality risk factors (44). In our sample, cardiorespiratory symptoms such as bronchiolitis, cardiac anomalies, asthma, and obstructive sleep apnea were present in 68.3% of the sample, indicating an increased risk of death.

Neurological disorders are also a health problem requiring the attention of caregivers and professionals (Table 3). Hypothalamic dysfunction may produce an exacerbated pyrexia response in infectious conditions. However, the absence of pyrexia has been observed in severe infections. Parents, caregivers, and health professionals need to be aware of the behavioral changes and other signs that may indicate problems in people with PWS. Late diagnosis may aggravate the associated diseases and increase the risk of death (45).

The prevalence of obesity-related metabolic diseases observed in this study were consistent with those reported in other studies (46, 47). Despite the severe obesity observed in the participants of this study (71.4%; Table 2), and in many other studies (47, 48), the prevalence of metabolic diseases in PWS was low when compared to typical subjects matched by the percentage of body fat. The causes of this lower prevalence of metabolic diseases are not clear; however, care should not be neglected. In our study, 65.9% of the sample had endocrine/metabolic anomalies such as hypercholesterolemia, hyperthyroidism/hypothyroidism, hypertriglyceridemia, and diabetes 1 and 2 (Table 3). In some cases of PWS in Brazil, the controversial decision to perform bariatric surgery has been taken (49, 50). However, the long-term results may not be effective considering the psychiatric comorbidities and intellectual disability that compromise the control of eating behavior.

Deviations in the spine, equinovarus foot and dysplasia are anomalies commonly observed in PWS and, in addition to hypotonia and obesity, they contribute to a change in body center of gravity, increasing the risk of falls and fractures (Table 3) (51, 52). This study reported three cases of surgical intervention related to fall fracture and two cases of surgery due to deviations in the spine (Table 4). Maintaining high levels of daily physical activity, and actively participating in physical activity may potentially contribute to strengthening muscles and bones (26, 53–57).

Behavioral problems are a common phenotype of PWS, and, in this study, we observed that anxiety was the main disorder reported by parents. Research has consistently shown that people with PWS resulting from UPD show a higher prevalence of psychiatric disorders than those resulting from DEL (58) and that behavioral problems increase with age (15). In this study, a positive correlation was observed between the age of the children and adolescents with problems with internalizing, attention, externalizing, and overall behavior. The findings reinforce the importance of behavioral psychological therapy to guide parents on how to manage children's behavioral problems and thus improve the family and social relationship interactions of the person with PWS (59).

The most used therapy by the participants of this study was speech therapy, followed by nutritional, psychological, hormonal, physical, and others (Table 3). Speech problems due to hypotonia and adenoid hypertrophy affect a large part of the population with PWS. Speech therapy can contribute to better social interaction (60, 61). However, it is worth mentioning that nutritional treatment, currently practiced by 58% of the sample (Table 3), should be practiced by all the participants of this study due their hyperphagic behavior and obesity (12). In addition to nutrition, physical activity has the potential to contribute to the improvement of behavior and body composition problems (53, 54, 56, 57, 62).

The complaints reported during the practice of physical activity in this study (Table 3) do not seem to be related to any dysfunction that completely prevents its practice. People with PWS are mostly hypoactive. However, if properly stimulated, they can become highly motivated to practice physical activity (53, 63). In this study, the main physical activity practiced by the PWS individuals was walking. Although the benefits of walking are well-known, it is important to ensure that the walking is done in at an intensity that produces the desired adaptations (improvements in cardiorespiratory fitness, body composition and a reduction in stress level) and that more elaborate exercise routines are implemented to amplify the neuromuscular (25, 64, 65) and cognitive gains (66).

Diabetes have been reported in 25% of adults and 12% of children with PWS (40, 67). In our study sample, diabetes was reported in 12% of the participants, with 7% presenting type 1 diabetes, which is uncommon for the syndrome. As our sample comprised a broad age range (1 month to 38 years old), this could have affected the relative frequency of diabetes. The prevalence of strabismus in the PWS population has been estimated to be about 60% (16). In our study, we reported two cases with strabismus. It is likely that this result could be associated with the small sample size or parent underreporting for this item in the questionnaire.

Parents reported a lower perception of quality of life in respect of the psychological, social relations, and environmental domains than the reference values for the Brazilian population (Table 4) (38). The results of this study are consistent with previous research that shows a deterioration in the perception of quality of life among caregivers of people with PWS when compared to parents of typical children and adolescents (30).

Interestingly, it was observed in the present study that the group of participants who used GH treatment had a lower level of externalizing problems and that parents tended to have a higher perception of life quality in the environment domain. As previously demonstrated in other studies, treatment with GH stimulates cranial-brain growth, increases lean mass and activity levels among children and young adults with PWS (22, 68). It is possible to infer that the gains from GH treatment result in an overall improvement in the health status of individuals with PWS, and a consequent concomitant increase in the parents' perception of quality of life. A previous study conducted in Brazilian PWS patients also reported the benefits of GHRT (69).

In this study, there were positive associations between age, and behavioral problems and health problems leading to hospitalization in those with PWS. As expected, families with higher incomes provided the greatest number of treatments; however, the number of treatments for those with PWS was not positively related to the quality of life of the parents.

The total number of hospitalizations showed a positive correlation with behavior problems. Despite the well-described clinical phenotypes exhibited among the individuals with PWS, it is possible to see a wide range of variation within them. DNA deletion in particular has been associated with seizure frequency (70) and brain structural and neural connection abnormalities s (71, 72). It is not unreasonable to assume that different deletion sizes could lead to variations in the number and severity of the physical and psychiatric comorbidities. Amaro et al. (53) reported that physical training reduces complaints of behavioral problems by the parents of children with PWS in a case study. Thus, it is possible to that an appropriate intervention program could promote a general improvement in health that leads to a reduction in behavioral problems (73, 74). However, more studies are needed to confirm this hypothesis.

It was hypothesized in this study that a greater number of treatments and therapies attended by those with PWS would be associated with a lower quality of life in their caregivers because of the constant pressure of medical visits resulting in increased stress. However, this hypothesis was not confirmed. The data from this study suggests that behavioral problems were the main element related negatively to the quality of life of the parents, either by the total behavioral problems presented or by the magnitude of the disorders.

Previous studies have shown a negative association between quality of life in PWS caregivers related to their child's age (29, 30), genetic subtypes (30) and motor performance (75). As previously discussed, parents of children with PWS may feel overloaded because of the continuous need to pay attention to the many aspects of their child's health, from very early childhood to older ages. Furthermore, behavior problems associated with PWS can lead parents to mental and emotional exhaustion (3), which contribute significantly to reducing their quality of life. Therefore, it is necessary to pay careful attention to the parents' health during all stages of their child's life, and provide instruction on how to cope with the syndrome as well as providing physical, psychological, and social therapies.

### Study Limitations

While the information presented in the study is novel as it relates to the Brazilian population, it has some limitations such as the small sample size; however, PWS is a rare disorder which makes gathering large samples difficult, and despite the small sample size, the study was able to provide a survey of the health problems in this population group and the impact of the condition on the quality of life of the parents and caregivers. In addition, data were collected through the parental report, which may lead to a potential interpretation bias. Furthermore, the participating families had a relatively high monetary income, which may not represent the overall Brazilian socio-economic reality. A final limitation is the absence of behavioral problem measures from those participants over 18 years old (19% of whole sample), because the youth version of BPM (BPM-Y) was not available in Brazil when this study was conducted.

## Conclusions

This is the first Brazilian study that characterized health problems and types of interventions in a sample of children, adolescents, and adults with PWS. Furthermore, this is the first study that evaluated the association between demographics, health, and behavioral problems in people with PWS and their parents' quality of life. Additionally, this study showed that health problems of the group were similar to those shown in previous research. An increased number of therapy and treatments for those with PWS was not associated with their parents' quality of life, while behavioral problems were negatively associated with parents' quality of life. Future studies should consider to what extent access to mental health services in this population determines the effects on the quality of life and mental health of individuals with PWS and their primary caregivers. There is clear need for multidisciplinary interventions for individuals with PWS, and health teams should not only focus on monitoring their patients, but also the mental health and quality of life of caregivers and parents.

## Data Availability Statement

The datasets generated for this study are available on request to the corresponding author.

## Ethics Statement

The studies involving human participants were reviewed and approved by Comitê de Ética e Pesquisa Universidade Presbiteriana Mackenzie. Written informed consent to participate in this study was provided by the participants' legal guardian/next of kin.

## Author Contributions

AA, LC, DR, and MT designed the study, drafted and wrote the manuscript, and revised and replied to the reviewers' comments. AA and AF performed the research and acquired the data. AA, LC, GR, and DR interpreted and analyzed the data. All authors contributed to the article and approved the submitted version.

## Funding

This research was supported by the Universidade Presbiteriana Mackenzie through doctoral scholarships, the Coordenação de Aperfeiçoamento de Pessoal de Nível Superior (CAPES) by supporting academic exchange through the Programa de Doutorado-sanduíche no Exterior (PDSE)—Proex number: 1133/2019.

## Conflict of Interest

The authors declare that the research was conducted in the absence of any commercial or financial relationships that could be construed as a potential conflict of interest.

## Publisher's Note

All claims expressed in this article are solely those of the authors and do not necessarily represent those of their affiliated organizations, or those of the publisher, the editors and the reviewers. Any product that may be evaluated in this article, or claim that may be made by its manufacturer, is not guaranteed or endorsed by the publisher.
